# Developing a theory-based multimedia intervention for schools to improve young people’s asthma: my asthma in school (MAIS)

**DOI:** 10.1186/s40814-020-00670-6

**Published:** 2020-09-02

**Authors:** Gioia Mosler, Katherine Harris, Jonathan Grigg, Liz Steed

**Affiliations:** 1 Centre for Genomics and Child Health, Blizard Institute, Barts and the London School of Medicine and Dentistry, Queen Mary University of London, 4 Newark St, London, E1 2AT UK; 2 Centre for Primary Care and Public Health, Barts and the London School of Medicine and Dentistry, 58 Turner St, London, E1 2AB UK

**Keywords:** Asthma, Self-management, Behavioural change, Adolescents, Young people, Intervention, Multimedia, Theory-based, Engagement, Gamification

## Abstract

**Background:**

Asthma control in adolescents is low with half of the young people in a London study identified as having suboptimal control when measured using the Asthma Control Test. Control of asthma symptoms can be improved by addressing barriers to good self-management, such as poor understanding of asthma and adherence to medication. The aim of this study was therefore to develop the My Asthma in School (MAIS) intervention for the improvement of asthma control and self-management in adolescents and to test its initial feasibility. The intervention intended to combine a strong focus on theory with a design specifically aimed to engage adolescents.

**Methods:**

The intervention development was based on previous qualitative and quantitative findings, and on guidelines from the Medical Research Council for the development of complex interventions. The COM-B (Capability, Opportunity, Motivation–Behaviour) model was applied to inform the design of intervention elements. Behavioural targets were identified from existing barriers to good asthma self-management and were then used to guide the development of engaging intervention elements, which were described using the Behavioural Change Technique (BCT) Taxonomy version 1. Adolescents were involved throughout this process. The MAIS intervention was tested in a feasibility phase in London secondary schools with adolescents aged between 11 and 13.

**Results:**

The complex school-based MAIS intervention comprised a first school visit from a theatre group, who conducted a workshop with all year 7–8 students and addressed peer understanding and attitudes to asthma. The second visit included four self-management workshops for adolescents with asthma, including games, short-films and role play activities. Forty different types of techniques to change behaviour were applied, totalling 163 instances of BCT use across intervention elements, addressing all areas of capability, opportunity and motivation. In this initial feasibility study, 1814 adolescents with and without asthma from nine schools received the theatre intervention visit; 23 adolescents with asthma from one of the schools attended the workshop visit. The intervention was found acceptable and engaging, and 91.4% of participants agreed that the workshops changed how they think or feel about asthma.

**Conclusion:**

This study demonstrates development and initial feasibility of a complex theory-based intervention, and how it can combine engaging media and interactive elements, to achieve a multi-directional approach to behavioural change. However more work is needed to assess the feasibility of trial processes, including recruitment and delivery format of the workshops.

## Key messages regarding feasibility

- What uncertainties about feasibility existed?

There was no information on the acceptability of the developed MAIS intervention and the feasibility of its delivery in the school context.

- What are the key findings about feasibility?

The delivery of the MAIS intervention is feasible within the school context, and both elements of the intervention received very positive feedback from participating children and young people.

- What are the implications of these results for the design of the main study?

The results of this feasibility study can inform the design and development of a randomised controlled trial.

## Background

Asthma is the most common chronic condition in children and young people in the UK [1]. For the majority of asthma patients, medications have the potential to control symptoms well [1]. Yet, there is evidence in the UK that almost half (49.6%) of young people (between 12 and 18 years of age) has suboptimal asthma control when assessed by the Asthma Control Test (ACT) [2]. Poor control frequently leads to unscheduled medical care with 30% of young people reporting an unplanned visit to a general practitioner (GP) in the past month [2]. UK also has a higher rate of asthma-related deaths compared to other European countries [3, 4]. Results from the school-based asthma project (SAP) [2] reported a lack of understanding of symptom severity. Of the young people identified with suboptimal asthma control, 42.4% judged their asthma to be either ‘well’ or ‘completely controlled’ [2].

Insights into why many adolescents do not have good asthma control are provided by qualitative findings from the SAP study. For example, reported barriers to successful self-management included forgetfulness, a lack of understanding of asthma and prescribed medication, as well as a fear of negative social interactions, such as teasing or bullying. These findings are similar to other studies which have reported forgetfulness as a barrier to adherence to asthma medication [5, 6] and desire to fit social expectations [5] as important influences on adherence and control.

Adolescence is a time of great physical, social and emotional change, resulting in age-specific needs. Adolescents with asthma therefore have different needs in relation to their chronic condition compared to smaller children or adults [7]. During adolescence, patients transition from predominantly parental management of their asthma to individual self-management. At this time, young people might question or change previously established routines, as they start to take more independent decisions about their health. Adolescence may therefore provide the opportunity to set up optimal behavioural standards for a young person which have the potential to last into adulthood [8].

Reviews of interventions in young people suggests that schools are effective locations to deliver asthma self-management interventions [9–13] which can improve outcomes, such as medication use and health-related quality of life, and can reach individuals who might not regularly access healthcare.

The need for a novel, theoretically informed intervention to improve young people’s attitudes and understanding of asthma, and subsequent self-management and symptom control, was identified. The My Asthma in School (MAIS) intervention was designed to achieve this. The intervention targeted young people aged 11–13 years, in order to embed the behavioural standards early in adolescence. This paper describes (i) the development of the MAIS in line with MRC principles and (ii) initial feasibility and acceptability testing of the intervention.

## Methods

### Intervention development of the my asthma in school intervention

**Fig. 1:**
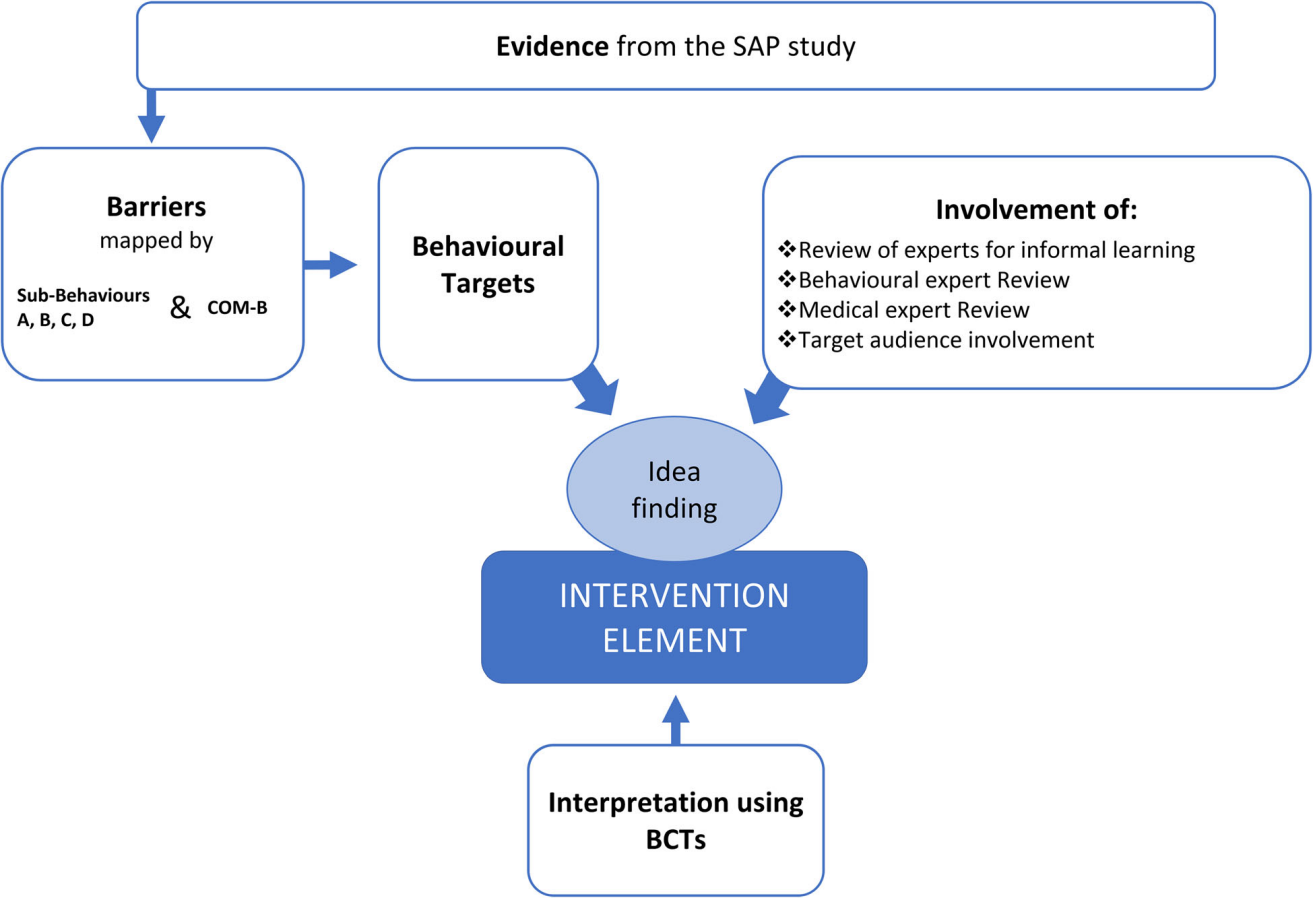
Overview over intervention development

The aim of the My Asthma in School (MAIS) intervention was to improve asthma control and self-management in adolescents. An overview of the intervention development is presented in Fig. 1 and described using the TiDieR framework (Supplement 1) and a logic model of the intervention (Supplement 2).

Intervention development followed MRC complex intervention guidance which suggests that complex interventions should be (1) evidence based and (2) theory based. The guidelines also state the importance of (3) involving of experts. Medical and behaviour change specialists were consulted throughout the development. In order to maximise engagement and commitment of adolescent patients, practical educational expertise was also included. (4) The involvement of patients and their community was central throughout all development steps. As part of this development phase, further feasibility work is recommended before formal evaluation in a trial context.

#### Evidence base

Systematic reviews suggest the need to improve self-management of asthma in the adolescent population [9–13]. However, it is important to understand the specific barriers to self-management and delineate separate behaviours within self-management. The SAP study [2] used qualitative methodology to understand what self-management meant to adolescents and where they were having difficulties. This directly informed the identification of target behaviours and specific areas to focus the intervention.

#### Theory base

The approach used for MAIS was the behaviour change wheel, with a focus on the theoretical domain framework and the COM-B model (Capability, Opportunity, Motivation–Behaviour) at its centre [14]. An important first step is to understand the behaviour (poor self-management) and break it down into its component target behaviours, here termed sub-behaviours. Based on previous literature [1, 15–17], six sub-behaviours to asthma self-management were identified and can broadly be described as:

A. Adherence to medication: This can be considered as whether one follows the prescribed medication regimen, but is interpreted here as encompassing not only the taking of medication but also factors influencing this, such as recognition of symptoms and understanding how common asthma medicines work.
B. Inhaler technique: Most young people with asthma regularly use an inhaler to administer their asthma medication. According to Asthma UK, up to 1/3 of people with asthma use their inhaler incorrectly and therefore get less of their required medication [18]. Spacers that can be attached to many regularly prescribed inhalers increase the intake of inhaled medication and can reduce the side effects of medication [19]. Spacers are however often considered by adolescents to be childish and are therefore often not used as prescribed [19–21].
C. Trigger avoidance: Asthma symptoms get worse when exposed to certain triggers. Triggers vary individually but can include pet hair or air pollution. Exposure to many different triggers can be avoided or reduced, and recognition of personal triggers and possible ways to avoid exposure are a crucial part of asthma management.
D. Emergency response: Knowing relevant steps to take during an asthma exacerbation is crucial for asthma sufferers. It was indicated by several participants of the SAP focus groups that they did not know what to do in an asthma emergency, and they could not advise others either.
E. Effective communication with healthcare professionals: It is essential for a patient to communicate quickly and effectively during a consultation with a healthcare professional, in order to receive the best possible care [1].
F. Empowerment to self-manage: In order for an individual to carry out a behaviour, they must feel they have knowledge, sense of control and confidence to carry out this behaviour. This is known as empowerment.

### Setting behavioural targets and mapping behavioural barriers

Within each sub-behaviour, the identified barriers from previous qualitative and quantitative data collections were mapped by two authors, one an expert in behaviour change. We used the COM-B model to categorise whether the barrier reflected limitations in an individual’s physical and/or psychological capability, their physical and/or social opportunity, and their automatic and/or reflective motivation. This allowed us to consider for each sub-behaviour which areas the intervention should target. As a next step, we then defined new targets to support self-management. Table 1 shows a summary of this work.

**Table 1:** Sub-behaviour, related barriers, organised by COM-B element, and resulting behavioural targets

Sub-behaviour	COM-B category^**a**^	Barriers to specific behaviours^**b**^	Behavioural targets in participants
**A) Adherence to medication**	**Capability** -Physical	Unpleasant side-effects (F and QN)	A1. Knowledge of how to manage side effects
	*-*Psychological	• Forgetfulness (F and QN) • Lack of knowledge and understanding regarding: (a) medication (F and QN), (b) side effects (F), (c) symptom severity (QN)	A2. Awareness of situations in which they could forget their medication A3. Knowledge of how different inhalers function A4. Knowledge what to do in case of side effects A5. Awareness of the differences between long-acting corticosteroid inhalers and short-term acting SABA inhalers A6. Comprehension of what is meant by well-controlled asthma A7. Appreciation of different asthma symptoms and symptom severity A8. Knowledge how a peak flow meter can be used to monitor symptom severity
	**Opportunity** -Physical	• Medication not available when needed: (a) misplaced inhaler (SABA or corticosteroid) (QN), (b) forgot SABA inhaler at home (QN), (c) inhaler expired (QN) • Inconvenient to take medication (F and QN)	A9. Ensure sufficient medication in different locations if appropriate A10. Awareness of the importance of reminders, prompts and cues, as well as the ability to set them up
	-Social	Social environment does not support taking medication: (a) not feeling comfortable taking inhaler at school (QN), (b) embarrassment (F), (c) reluctance to use in public (F)	A11. Competent talking about asthma A12. In participant’s peers: understanding what it means to live with asthma
	**Motivation** -Automatic	Uncomfortable with medication—unspecified (QN)	A13. Awareness of the importance of medication adherence
	-Reflective	• Belief that medication is ineffective (QN) • Inhaler efficacy (F), in particular SABA efficacy (QN) • Belief that medication not required: (a) corticosteroid inhaler, (b) SABA inhaler and (c) tablets (QN) • Fear of reliance (F) • Use other medication instead: (a) SABA instead of corticosteroid inhaler (QN), (b) corticosteroid inhaler instead of SABA (QN), (c) different medication instead of tablets (4 tablets) (F) • Do not want to take the medication: (a) corticosteroid inhaler, (b) SABA inhalers, and (c) tablets (F & QN) • Laziness (QN) • Inhaler apathy (F) • Excuse to miss class (F)	A13. Awareness of the importance of medication adherence (s.a.) A3. Understanding how different inhalers work (s.a.) A5. Awareness of the differences between long-acting corticosteroid inhalers and short-term acting SABA inhalers (s.a.) A6. Comprehension what is meant by well-controlled asthma (s.a.) A7. Appreciation of different asthma symptoms and symptom severity (s.a.)
**B) Inhaler technique**	**Capability** -Psychological	Lack of knowledge: (a) inhaler technique (F), (b) spacer usage (QN)	B1. Proficiency in the correct inhaler technique B2. Understanding how a spacer acts and what the benefits are of using a spacer
	**Opportunity** -Physical	Limited spacer usage (QN)	B3. Knowledge about how to acquire a spacer
	*-*Social	Spacer is embarrassing [19–21]	B4. Proficiency in problem-solving skills for managing emotions and difficult social situations related to spacers
	**Motivation** -Automatic	Spacer is embarrassing, see also above, social opportunity	B4. Proficiency in problem-solving skills for managing emotions and difficult social situations related to spacers (s.a.)
**C) Trigger avoidance**	**Capability** -Psychological	Lack of knowledge of which triggers exist, what triggers do	C1. Knowledge about different triggers and how to mitigate their effect
	**Opportunity** -Social	Lack of social support in reducing effect of triggers (esp. for exercises) (F)	C2. Proficiency in problem-solving skills for difficult social situations related to triggers
**D) Emergency response**	**Capability** -Psychological	Lack of knowledge about how to respond to an asthma emergency (F)	D1. Skilled in asthma emergency response
**E) Effective communication with healthcare professionals**	**Opportunity** -Physical	Lack of consistency of care (F)	[Possible solutions lie outside planned school-based intervention]
	-Social	Perceived problems with the communication of healthcare professionals (F) including: (a) they do not feel fully informed by healthcare professionals, (b) communication from healthcare professionals is confusing, (c) feeling that the healthcare professional did not respond adequately to their concerns	E1. Appreciation of good communication with healthcare professionals E2. Understanding of the role of a healthcare professional E3. Proficiency in methods of preparation for a visit with a healthcare professional
**F) Empowerment to self-manage**	**Capability** -Psychological	Belief of people with asthma that they do not know enough about their own condition (F), including (a) belief they are lacking general skills and information (F), (b) not knowing what causes asthma (F)	F1. General understanding about asthma A11. Competent talking about asthma (s.a.) F2. Knowledge why someone develops asthma
	**Opportunity** -Social	• Perceived lack of understanding by non-asthmatics (F): (a) feeling not taken seriously, (b) people without asthma are not listening, (c) asthma is not talked about, (d) fear of being bullied or ridiculed, (e) peer awareness • Perceived stigma (F)	F3. In participant’s peers: awareness and acceptance of asthma F4. Proficiency in problem solving skills for difficult social situations related to (a) ridicule, (b) communicating symptoms, (c) responsibility to self-manage
	**Motivation** -Reflective	Embarrassment and not wanting to address asthma: (a) embarrassment, (b) not wanting to accept and deal with asthma	F5. Acceptance that asthma is part of them F6. Appreciation that asthma is very common F7. Appreciation of possibilities and limitations of a life with asthma F8. Proficient in methods aimed to change their asthma themselves, including (a) where to get support, (b) how to break down a problem to find the most effective solution, (c) SMART goal setting technique F4. Proficiency in problem-solving skills for difficult social situations related to the responsibility to self-manage (s.a.)

^a^Not all sub-behaviours show barriers within each COM-B category

^b^(F) evidence from SAP focus groups, (QN) evidence from SAP questionnaire

For five of the six sub-behaviours, psychological capability emerged to be one of the most commonly mapped COM categories. For example, barriers to sub-behaviour: (A) Adherence to medication, such as ‘Forgetfulness’ and for sub-behaviour (D) Emergency response, the ‘Lack of knowledge about how to respond to an emergency’. Many barriers were also mapped to the COM category of social opportunity across different sub-behaviours. Examples include barriers to sub-behaviour (C) Trigger Avoidance: ‘Lack of social support in reducing effect of triggers’, or barriers to (F) Empowerment to self-manage, such as a ‘Perceived lack of understanding by non-asthmatics’. These findings directly informed the focus of the developed intervention.

### Developing the intervention format and elements

#### Intervention format

As psychological capability was a key target area for the intervention, including improvements on knowledge and understanding of asthma; workshops for young people with asthma aged 11 to 13, supported with resources such as apps and information booklets, were felt to be an appropriate form of delivery. Workshops have been used successfully by similar interventions and allow for tailoring and engagement of participants [9]. They furthermore are highly suitable to the school environment. Due to the risk of a high dropout rate from repeated visits, based on experience with previous work in schools, a 1-day workshop with four parts was developed.

The evidence (shown in Table 1) suggested that social opportunity, i.e. involving the peer group of young people with asthma, was another important target area to support self-management. Theatre workshops are a medium which has previously been proven effective in addressing attitudes in young people [22] and are therefore considered a novel intervention to target the peer environment. They can also be delivered to large numbers of individuals and can be accommodated with relative ease in the school context.

#### Developing engaging and effective intervention elements

To ensure engaging and appropriate workshop content, iterative input was gained from:

i) Over 50 young people with asthma between 10 and 16 years of age during over 40 visits to the waiting rooms of two paediatric asthma clinics in London hospitals. A further eight focus groups in schools were used to receive input on intervention elements.
ii) An outreach and learning team of experts with dual backgrounds in research and research communication who worked directly with the university’s science education centre, Centre of the Cell. This is a science centre specialising in the development and delivery of engaging workshops, shows and video games with a focus on medical topics for young people [23]. Ideas about how to address identified behavioural targets within the intervention were regularly discussed between the outreach and learning team and the Centre of the Cell team of science communicators.
iii) Multidisciplinary experts from the fields of behaviour change, respiratory conditions, teaching, and paediatrics
iv) A National Institute for Health Research (NIHR) lay advisory panel, a group of lay people organised by the NIHR met with the research team and discussed various aspects of the project. Elements of the study were also presented to the public at interactive stalls during six science and community festivals, such as the annual QMUL festival of communities.

Workshop content was reviewed in regard to age-appropriate design and language, as well as theoretical appropriateness according to behaviour change theory. Ideas were then tested and refined.

Work on the ‘In Control’ theatre provocation was undertaken by GLYPT (Greenwich and Lewisham Young People’s Theatre) Tramshed, who are experts of performance arts aimed at changing attitudes in adolescents and have in the past worked with young people about gang violence. The MAIS team collaborated with GLYPT on the development of the theatre workshop and organised training about the medical aspects of asthma for the theatre cast.

### Prototype intervention and categorisation of the intervention elements using BCTs

#### Prototype intervention for testing

The developed intervention involves two school visits (Table 2). The first visit is by the GLYPT theatre group, performing ‘In Control’ to the whole school year groups 7 and 8 (11 to 13 years of age) and addresses acceptance of asthma and understanding in both young people with asthma and their peers. Following a 40-min performance, there is a facilitated discussion with actors both in role then out of role, working through the main concepts of asthma acceptance within the characters and for the audience. During the second school visit, two members of the MAIS team facilitate four consecutive 1-h workshops to young people with asthma from year groups 7 and 8, with additional support from a member of the research team. During the scripted workshops, participants learn about asthma and the four asthma self-management sub-behaviours.

**Table 2:** Intervention and elements with type of activity and behavioural change techniques (BCT) [24]

	Part of intervention	Element	Targets to support self-management addressed^a^	BCTs^b^
Visit 1	**Theatre** (whole year groups)–90 min	In control, interactive theatre	A11; A12; F3; F7; F5	3.1; 5.1; 5.3; **5.2**; 6.1; 6.3; 9.1; **12.2**; 13.2
Visit 2	**Workshop 1** (60 min): What is asthma? Who has asthma?	Asthma is… group quiz	F5; F6; A11	8.1
		Wall of fame, hands-on sorting game	F5; F6; F7	6.2; 9.1.
		Asthma balance, group competition	F1; A11; F2	4.3
		Giant airways, hands-on activity/demonstration	A3; A5; A13; F1	4.1; 4.2; 5.1; **5.2**; 11.1
		Define asthma, partner discussion	A11	8.1
	**Workshop 2** (60 min): symptoms and triggers	Symptoms intro, discussion and demonstration	A7	5.1; 4.1
		GP consultation, interactive firm clips and discussion	A3; A5; A11; A13; E1; E2; E3	**1.2**; 1.4; 4.1; *4.2*; 5.1; 5.2; 5.3; 6.1; 7.1; *9.1*; *11.1*; 11.3; 12.5; 13.2; 16.3
		Peak flow interactive, demonstration	A6; A8	2.6; **4.1**; 6.1; 12.5
		Good control discussion	A6	**4.1**; 6.1; *8.1*
		Trigger map, hands-on activity and discussion	C1; C2	3.2; **4.1**; 4.2; 5.2; 5.3; 7.5; 11.2; 12.1; 12.2; 12.3; 7.1; 12.5
		Asthma dash board game	F1; A11; A2; A3; A5; C1; C2; A13; F5; F8	During game: *2.2*; *3.1*; *4.1*; **5.2;** 5.3; *6.2*; *7.1*; *7.5*; *8.1*; *9.3*; *10.3*; *10.11*; *14.2*; Description of game rules: *4.2*; 5.1; *11.1*; *11.2*; *12.1*
	**Workshop 3** (60 min): medicines and emergencies	Medication myth buster, group quiz	A3; A4	5.1; **9.2**; 11.1
		Inhaler shuffle, group quiz	A5	5.3; 11.1
		The big mouth, hands-on interactive game	B1; B2; B3	4.1; 4.3; 5.1; **5.2;** 6.1; 12.5; 11.1
		Puffer partners, demonstration and training	B1; B2	2.2; 4.1; 6.1; **8.1**
		Emergency role play	D1	1.2; 3.2; 4.1; 5.1; 8.1
	**Workshop 4** (60 min): support network and taking control	Breaking the jump, short movie	F5; F7; F8, A11	4.1; *4.2*; 5.1; *5.2*; 5.3; 6.1; 9.1; 12.1; **13.2**
		Support target, discussion and writing	F8	3.1
		The third option, short movie	F8	***1.2;*** *4.1; 5.1;* 5.2; *5.3;* 6.1; *7.1;* 9.1; *9.2; 9.3*
		What can I do? Facilitated discussion	A10	3.1; 7.1; 12.1
		Asthma solution, partner discussion	A11; B4; F4	1.2; 3.1; 5.3; **9.2**
		Setting goals, facilitated discussion and writing	A10; F8	1.2; 1.3; 1.4; 1.9; 7.1
	**Booklet**—to take home (introduced during workshops)	Booklet	B3	4.1; 4.2; 5.1; 9.2; 11.1; 11.2; 12.1
	**Toolbox**—to take home (introduced during workshops)	Boost, mobile game app	Sustained learning	4.1; 4.2; 5.1; 5.3; 7.1; 7.5; 11.1; 11.2; 12.1; 12.3
		Asthma action plan		4.1
		Asthma dodge, mobile game app	A3; A5; A7; A13	4.1; 4.2; **5.2**; 5.3; 6.1; *7.1*; 7.7; *8.1*; *8.3*; *8.7*; *10.3*; 11.2; 12.2; 12.3; *13.2*; *14.4*; *14.5*
		GP calling card	B3	7.1
		Peak flow chart		12.5

Indirect BCTs are presented in cursive, overarching BCTs in bold

^a^Full descriptions of behavioural targets are presented in Table 1

^b^Full descriptions of BCT codes are presented in Supplement 3

#### BCT categorisation

All agreed elements of the intervention were coded using the Behavioural Change Taxonomy (BCT) version 1 by Susan Michie [24]. The taxonomy lists 93 behavioural change techniques which are categorised into the following groups: (1) goals and planning, (2) feedback and monitoring, (3) social support, (4) shaping knowledge, (5) natural consequences, (6) comparison of behaviour, (7) associations, (8) repetition and substitution, (9) comparison and outcomes, (10) reward and threat, (11) regulation, (12) antecedents, (13) identity, (14) scheduled consequences, (15) self-belief, (16) covert learning. Two coders independently coded all intervention elements, and any discrepancies were resolved through discussion. Table 2 shows the draft intervention with each element coded for BCTs and standards to support self-management.

Elements of the workshop include a short movie around problem solving and goal setting, role play to learn about appropriate emergency response and a giant airway to demonstrate air flow and the effects of different medicines. An information booklet and toolbox are handed out at the end of the workshop day. Elements of the intervention are designed to function as standalone activities or be combined for a tailored approach and have therefore been coded for BCTs separately. One technique however relates to the whole intervention. This technique is ‘credible source’, referring to the trained professionals, who are organising and facilitating the intervention. The use of protagonists in media elements of the intervention meant that several BCTs were applied indirectly, through avatars. These indirect BCTs have been indicated in cursive in Table 2.

**Fig. 2:**
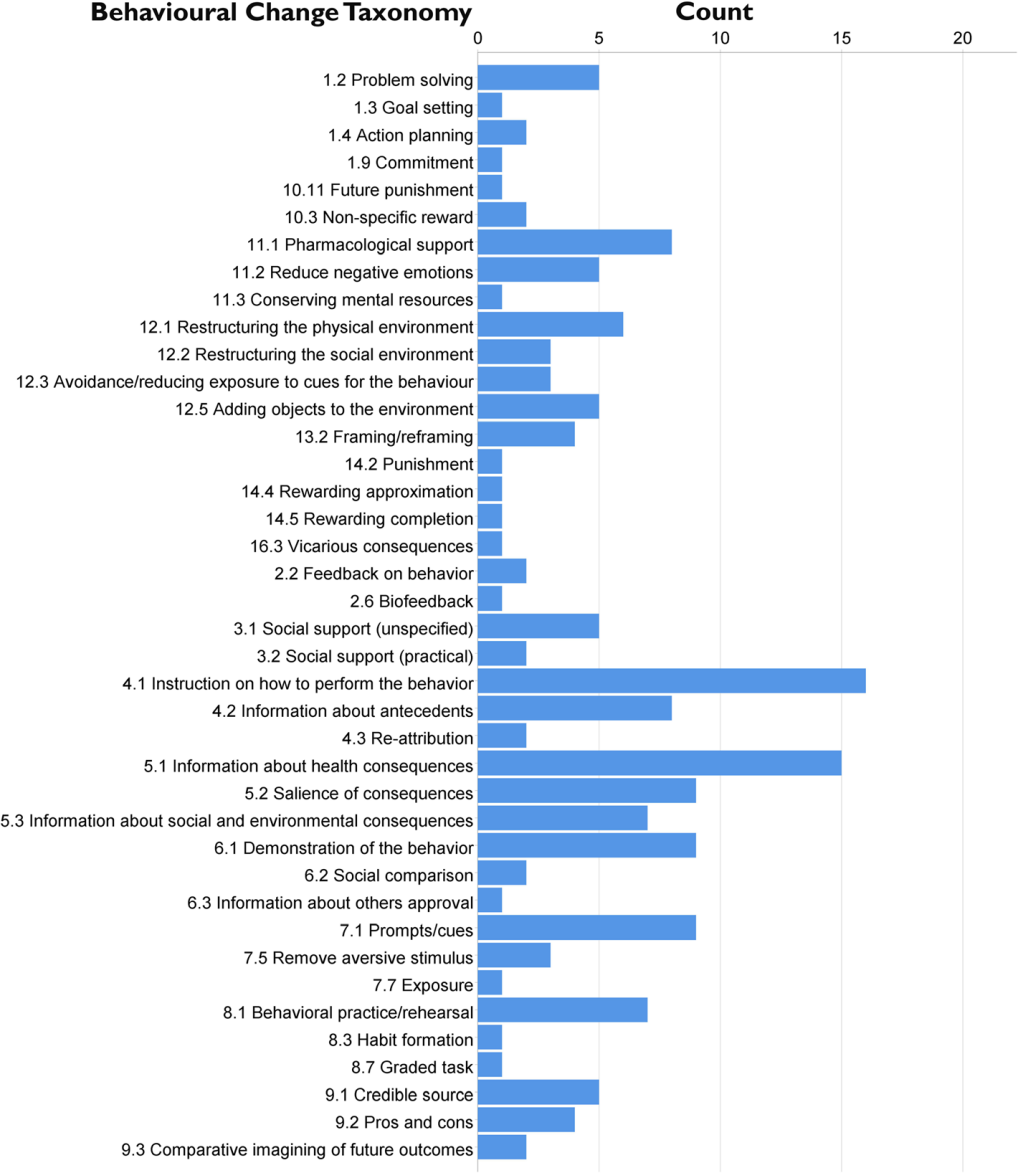
Frequency of applied behavioural change techniques, according to the BCT taxonomy by S. Michie

In total, 29 intervention elements were separately coded, including single elements from the workshops and the toolbox. One hundred sixty-three behavioural change techniques (BCTs) were identified for the intervention, including 40 different BCTs (Fig. 2). In line with our objective to improve asthma self-management skills, the BCT applied most frequently within the intervention was 4.1, ‘Instruction on how to perform the behaviour’ with sixteen applications and nine applications of 6.1, ‘Demonstration of the behaviour’ and 8.1, ‘Behavioural practice/Rehearsal’ with seven applications.

The number of BCTs applied within any element of the intervention was between 1 and 18, which reflects how many different approaches to behavioural change are included within the intervention. The highest number of different BCTs has been coded for two games: ‘Asthma Dash’ board game (workshop 2) with 18 BCTs, and ‘Asthma Dodge’, mobile app game (toolbox) with 17 BCTs. Both games encompass several sub-behaviours including avoiding triggers and adherence to medication. They both put a focus on the reinforcement of behavioural change through 5.2, ‘salience of consequences’. Other BCTs that were coded for both elements include 7.1. ‘prompts/cues’, 8.1. ‘behavioural practice/rehearsal’, and 10.3. ‘non-specific reward’. Further details on the BCT coding are provided in Supplement 3.

### Feasibility phase

The feasibility phase was conducted to:

- Test practicability and feasibility of delivering the intervention, especially within the school context;
- Understand acceptability of and participant’s engagement with the intervention;
- Find out if the topic areas and elements addressed in the intervention had been understood;
- Gather general feedback about the intervention with a view to refinement.

#### Design

A pre-post study design to assess feasibility, acceptability and knowledge gained through both workshop and theatre visits.

#### Recruitment of schools

All schools who had previously worked with the study team on the school asthma project (between 2015 and 2018) or who had experience working with the GLYPT theatre company were invited to participate in the feasibility study (*n* = 50). Participants from London secondary schools who took part in the SAP were recruited from different year groups for the MAIS. If a school agreed to be part of the feasibility study and receive the theatre workshop, they were then offered the 1-day workshops

#### Inclusion criteria

Theatre performance:

- Children aged 11–13

Asthma workshops:

- Children with asthma as specified by the school asthma register
- Children who had attended the theatre production

#### Exclusion criteria

Theatre performance:

- Children younger than 11 or older than 13.

Asthma workshops:

- Any child not on the school asthma register
- Any child who did not attend the theatre production

#### Consent

##### Theatre performance

For the theatre performances, each school gave consent at a school management level. This form of gate keeper consent is common when interventions are delivered at a cluster level (i.e. in a school) and was considered appropriate, as the theatre does not identify individuals with asthma or discuss medical details about the condition, but focuses instead on perceptions around attitudes to health in all adolescents.

##### Workshops

For the workshop visits, given previous experience that parental opt-in consent resulted in a drastic reduction of participants and given the low risk of the proposed intervention, parental opt-out consent and young people’s assent were collected.

##### Procedures

For economic reasons, the theatre part of the intervention was undertaken as a performance tour with all performances delivered by a team of young actors during three consecutive weeks. The workshop day was then offered to the schools who received the theatre performance for adolescents with asthma.

##### Evaluation

Given the different recipients of the theatre visit (all young people) and the workshops visit (young people with asthma), the feasibility of these two school visits was assessed separately.

Feedback for the theatre performances was collected in a short questionnaire handed out and completed by the audience in a postcard-sized format directly after the end of the performance. The questionnaire included questions related to the enjoyment of the production itself, attitudes to asthma and attitudes to general health issues. There was room for free text comments about the performance.

The impact of the workshops on asthma knowledge was assessed using a previously developed questionnaire. Additional feedback questions after the workshops asked about acceptability and engagement. The research team also gathered evidence about practicability and general feasibility of the intervention.

## Results

### Feasibility phase results

Of the 50 schools invited to participate, nine schools agreed to receive the theatre performances. Twenty-two performances were arranged over a 3-week tour to ensure all 1814 young people in year groups 7 and 8 in these schools were targeted (mean 2.4 visits per school). The school visits for the theatre tour were arranged in a first step. Once the tour schedule was agreed, the research team then provided schools with the additional opportunity to participate in the workshops. Only one of these 9 schools accepted the additional offer of 1 day of asthma workshops. Given the low uptake of workshop visits, this two-step recruitment strategy was reviewed, as described in the ‘Discussion’ section.

### Theatre provocation

Feedback forms on the theatre performance were filled out by all attending 1814 young people between 11 and 13 years of age. Feedback results are presented in Table 3.

**Table 3:** Feedback for the ‘In Control’ theatre as part of the intervention feasibility

1. How enjoyable was the performance?				
Not at all	Hardly	Somewhat	Very	Completely
0.6% (11)	0.7% (13)	7.3% (131)	38.5% (693)	52.8% (950)
2. Did watching ‘In Control’ change how you think or feel about living with asthma?				
Not at all	Hardly	Somewhat	Very	Completely
7.2% (129)	8.2% (146)	26.9% (480)	33.3% (593)	24.4% (434)
3. I want to help people with asthma			62% (1122)	
4. I feel I understand people with asthma better			65.8% (1191)	
5. My opinion about asthma has not changed			12.6% (228)	
6. I feel motivated to look after my own health better			52.7% (953)	
7. I feel people should talk about health issues more			53.9% (975)	

Numbers in brackets represent number of participants. For questions 3 to 7, participants choosing ‘Yes’ as an answer are presented (options: Yes/No)

The general feedback was very positive, and 91.3% of the audience said the theatre performance ‘In Control’ was very or completely enjoyable. 84.6% of the young people said that the performance also at least ‘somewhat’ changed how they think or feel about asthma. More specifically, more than 60% of the participants felt the performance made them want to help people with asthma (62%), and the performance made them understand people with asthma better (65.8%). The theatre provocation also had an impact on the participant’s wider health perceptions. Over half of the young people (52.7%) said the performance made them want to look after their own health better, and 53.9% said that the performance made them feel that people should talk more about health issues.

Examples of 155 free text comments from children with and without asthma included: ‘I want to make people happy with asthma’, ‘Never laugh at people who have asthma (or any other healthy issues)’, ‘Should help my family with asthma’, ‘I wasn’t comfortable with my asthma until now’, and ‘I think that I should bring in my asthma pump and tell a teacher’.

The feasibility of including the performance as part of the intervention was reviewed between actors, the MAIS team, and GLYPT Tramshed half-way through the theatre tour, as well as after the last performance. Feedback from the actors was that there should be a limit of about 90 children for each performance whenever possible. The performances had on average 83 students attending. A number of up to 90 children allow to have the audience seated in a full circle around the stage with seats only two rows deep. This seating arrangement makes it easier to engage with the audience, compared to a classic set up with a frontal stage and multiple rows.

### Workshops

When the schools were asked if they would like to receive a day of asthma-related workshops in addition to the planned theatre visit, one of the nine schools agreed to accommodate the workshops. The asthma register of that school identified 23 pupils with asthma in years 7 and 8. All of these engaged and completed the workshops. Nineteen participants completed the questionnaire as shown in Table 4. Fifteen of the 19 participants agreed or strongly agreed that the workshops were fun or interesting. Seventeen out of 19 participants also said the workshops changed at least somewhat how they think or feel about asthma and what it means to live with it. When asked to give examples of what changed, answers included ‘the fact that you always have to keep your inhaler with me, I didn’t think that this was serious before’, ‘I learnt how to handle certain situations’ and ‘to understand how to use inhaler better’.

**Table 4:** Feedback for the workshops as part of the intervention feasibility study

1. Did the workshops change how you think or feel about asthma and what it means to live with it? *n* = 19				
Not at all	Hardly	Somewhat	Very	Completely
1 (5.3%)	1 (5.3%)	6 (31.6%)	6 (31.6%)	5 (26.3%)
2. Were the workshops fun or interesting? *n* = 18				
Strongly disagree	Disagree	Uncertain	Agree	Strongly agree
0	1 (5.3%)	3 (15.8%)	8 (42.1%)	7 (36.8%)

Numbers in brackets represent percent of participants

The change in number of participants who correctly answered knowledge questions about asthma pre and post workshops are presented in Table 5. Generally, asthma knowledge was quite poor before the workshops and improved after the workshop. Knowledge of why a spacer should be used was particularly low before the workshops, with only 4 participants giving the correct answer. The highest increase from 6 to 12 participants was recoded for the question why people develop asthma. All general knowledge areas were correctly answered by at least 66.7% of participants after the workshops.

**Table 5:** Before-after questionnaire to assess knowledge about asthma in the feasibility study

Knowledge questions	Before, *n* = 19	After, *n* = 18
General asthma knowledge	**Correct before (%)**	**Correct after (%)**
How many young people have asthma	9 (42.3)	14 (73.7)
There are ways to reduce the effect that asthma has on life	11 (52.4)	15 (75)
Why do people develop asthma	6 (28.6)	12 (80)
Common symptoms of asthma	17 (85)	19 (90.5)
Asthma does not require treatment	17 (81)	19 (90.5)
Inhalers do not work if you do not feel them working	7 (33.3)	15 (71.4)
Spacers	**Correct before (%)**	**Correct after (%)**
Why should a spacer be used	4 (20)	14 (66.7)
Emergencies	**Correct before (%)**	**Correct after (%)**
I know what to do when someone has an asthma attack	9 (56.3)	17 (94.4)
When is emergency care needed	6 (42.9)	12 (70.6)
Triggers	**Median [mean] before**	**Median [mean] after**
Recognised triggers out of list of 10	6 [6.3]	10 [8.7]
Differences SABA and corticosteroid inhalers	**Median [mean] before**	**Median [mean] after**
Correctly identified inhaler statements out of 6	2 [1.6]	4.5 [3.9]

Post workshop reflections from the team suggested that the workshops were feasible and acceptable both to students and teachers who all responded positively to being part of the study. It was perceived that a group of 23 in the workshop was too large to maintain the intended interactivity. After discussing possible group sizes with Centre of the Cell learning experts, a maximum number of 15 was agreed for future workshops. Workshops 2 and 3 ran for 75 min which was reported as too long. It was therefore decided to reduce the number of iterations of some included activities, as well as change two group work activities into briefer partner activities. The preparation and logistics of the workshop were considered feasible.

The overall set up of the intervention in two visits was also reviewed and was judged to be feasible.

## Discussion

### Summary of findings

The development of the MAIS intervention demonstrates the benefit of applying a systematic and theoretical approach to intervention development and allows a thorough translation of barriers to self-management of asthma into new behavioural targets.

Use of a behaviour change taxonomy to code intervention elements ensured that the most relevant techniques were used and resulted in a rich and varied approach to support behaviour change. The use of different entertainment media and gamification, with media-based or gamified elements, also resulted in an engaging intervention, which was well received. Both elements of the intervention resulted in very positive feedback from the young people involved. 91.3% of the theatre audience said the performance ‘In Control’ was very or completely enjoyable, and 94.7% of workshop participants agreed the workshops were fun or interesting. Feedback by actors and the theatre company was positive. Running the theatre as a tour with all performances delivered by a team of young actors during three consecutive weeks worked well. Some small changes were undertaken to the intervention after the feasibility study, primarily in terms of workshop length, and implications for future trial procedures were apparent. Comments received from the actors about ideal audience size will furthermore inform future delivery.

The feasibility phase showed that the intervention content is acceptable and practicable in its proposed form, and that it has the potential to change attitudes and understanding towards asthma. Yet, it did highlight some issues with regards to the workshops. Primarily this related to the recruitment strategy, which offered the workshops to schools after they had been booked into the theatre tour. That meant teachers had already started to make arrangements for the theatre visit and would have needed to change plans in order to also accommodate the workshops. This additional commitment may have been a barrier to accepting the workshop visit. Future qualitative research will explore such barriers in more detail and consider changes to the recruitment strategy, for example by offering theatre and workshops elements together.

### Comparison to existing evidence

While interactive media is becoming increasingly common in behavioural change interventions, the assessment of these approaches is still in its infancy. The approach of using interactive media in this study was built on some initial positive findings that indicate potential benefits of using media for psychosocial, behavioural change or symptom relief [25]. It is clear that using, for example, serious games or gamification in an intervention has a great potential to engage participants through a rich sensory experience. Furthermore, a driving factor for behavioural change, recognised across different theories, is motivation to change the behaviour. Games have proven to be a very effective way to improve the motivation for a behaviour [26]. The MAIS intervention development indicates that theatre, workshop media and gamified elements of an intervention can not only work through their motivation potential, but also have the capacity to build skills and social opportunity.

An interesting finding when developing the intervention was recognising a distinction between directly applied BCTs and an opportunity to allow for indirect approaches to behavioural change within media-based approaches. Games, films and theatres often use an avatar with which the player or audience identifies in order to carry and receive behavioural messages or to perform behaviours on their behalf. Currently it is not clear whether there is a differential effect of indirect and direct BCTs, but recognising the difference is an important step towards this. It may be hypothesised that applying a behavioural change technique indirectly can help to reach patients who may consider a more direct approach too intrusive or may not be ready to change. It should furthermore be noted that a larger number of BCTs may not be a direct reflection of how effective an element might be in improving self-management behaviour, rather the appropriateness of the BCT for the underlying aim may be more important.

### Strengths and limitations

An important strength of the study is the robust development of the intervention. Having a feasibility phase in the real world also allowed for refinement of the intervention and alerted us to questions such as the best model of delivery, particularly of workshops. The inclusion of many different intervention elements, all using appropriately selected techniques to change behaviour, also has ensured the intervention is engaging.

There are however some limitations of the current study. In relation to the intervention, it mainly focuses on group work and leaves less time for a more personalised approach, although during the fourth part of the workshop young people identify their own social support network and use individual problem solving and goal setting techniques. The use of primarily group work is considered relevant given the importance of social opportunity to support self-management in young people with asthma; however, it remains to be seen whether greater tailoring or more individual work could be helpful. The main elements of the intervention are also delivered within a short time frame (2 days) rather than over a period of weeks, meaning practice of learnt skills within the intervention period is limited. This was decided in order to avoid drop-out over time and make delivery more feasible within a school setting where disruption on multiple days may be difficult. Elements such as apps and information materials were provided to the young people when leaving the workshops however to foster a sustainable effect.

The objective of the current study was intervention development, and therefore at this stage effectiveness data both on clinical outcomes or process measures such as change in behaviour was not possible; however, this would be the objective of future studies given the current findings of acceptability and feasibility.

Similarly, the design of the feasibility assessment particularly of the theatre provocation relied only on post-performance questionnaires and therefore does not account for baseline attitudes or understanding and therefore cannot reflect the degree of change. Assessment of the workshops did however involve baseline assessments and hence could show change, although the lack of a previously validated tool is a limitation.

The recruitment strategy was also problematic with only one school receiving workshops, so results may not be representative for other schools. A pilot study which not only confirms intervention acceptability but examines trial procedures would therefore be helpful before a full effectiveness study.

## Conclusions

The use of theory (including the MRC (Medical Research Council) guidelines for the development of a complex intervention and the COM-B model for behavioural change) together with expert and user input can lead to an acceptable and feasible intervention. This approach combined with an innovative and varied format including multimedia helped to ensure the intervention is interesting and engaging.

Consideration however should be given to how schools are offered both components of the intervention, i.e. theatre and workshop visits to the school to ensure uptake to both. Further evaluation is also needed on how the intervention can be delivered at scale.

The MAIS intervention will now be assessed with young people between 11 and 13 years of age in a pilot cluster randomised control trial (RCT) with a view to improvement in adherence behaviour, asthma control, quality of life and health care use.

## Supplementary information

**Additional file 1:.** Supplement 1: TiDieR Framework

**Additional file 2:.** Supplement 2: Logic model

**Additional file 3:.** Supplement 3: Elements of the intervention, behavioral targets related to them, as well as BCTs addressed by the element

## Data Availability

The datasets used and/or analysed during the current study are available from the corresponding author on reasonable request.

## Abbreviations

ACT: Asthma Control Test
BCT: Behavioural Change Technique (taxonomy)
CLAHRC: Collaboration for Leadership in Applied Health Research and Care
COM-B: Capability, Opportunity, Motivation–Behaviour
GLYPT: Greenwich & Lewisham Young People’s Theatre
MAIS: My Asthma in School (intervention)
MRC: Medical Research Council
NIHR: National Institute for Health Research
QMUL: Queen Mary University of London
RCT: Randomised Control Trial
SAP: School-based Asthma Project
TIDieR: Template for Intervention Description and Replication (checklist)
