# Supplementary material for: Developing a theory-based multimedia intervention for schools to improve young people’s asthma: my asthma in school (MAIS)
Source: Pilot Feasibility Stud. 2020 Sep 2;6:122. doi: 10.1186/s40814-020-00670-6 (PMC7465390; doi:10.1186/s40814-020-00670-6)
Supplement: Supplementary file 1 — Additional file 1:. Supplement 1: TiDieR Framework [file 40814_2020_670_MOESM1_ESM.docx]

## Supplement 1: TiDieR Framework

| **Item No** | **Item** | **Description** |
| --- | --- | --- |
| **Brief name** |  |  |
| 1 | Provide the name or a phrase that describes the intervention | Multifaceted theory-based self-management intervention to improve adolescents’ asthma control. Short title: ‘My Asthma In School’ programme |
| **Why** |  |  |
| 2 | Describe any rationale, theory, or goal of the elements essential to the intervention | It was shown in a literature review that there was a significant need to improve self-management of asthma in the adolescent population. Research was then conduced to identify the key determinants (both behavioral and environmental) of this goal. 6 key behaviors to asthma self-management were identified: a) Adherence to Medication, b) Inhaler Technique and Spacer Usage, c) Trigger Avoidance, d) Emergency Response, e) Effective Communicating with Health Professionals, f) Empowerment to Self-Manage.  As a next step, each behavior was mapped according to the capabilities, opportunities, motivations and behavior (COM-B) framework and subsequently developed interventions to target specific COM elements for each behavior with use of behavior change techniques according to the Behavioral Change Taxonomy (BCT)[15]. Barriers to each behavior were translated into behavioral targets that were to be set as part of the intervention.  The intervention is planned to be implemented in schools. The intervention comprises of the theatre and following theatre workshop for the whole year group, which will be delivered in the school during one morning. 4 workshops for children with asthma of the same year group are delivered within one week of the theatre.  The intervention will initially be delivered as a pilot to a randomised control trial. The trial will have three arms with schools randomly allocated to either a) a full intervention, b) theatre only, or c) the control arm. A power calculation has been undertaken to identify the number of schools needed, which identified that the pilot would need 20 schools with altogether 360 children with asthma to take part. The project has worked with 24 partner schools in the past who will be offered participation in the intervention trial.  The main aim of the intervention is to improve asthma control and self-management in young people with asthma, secondary aims are to improve the environmental context this is managed in through peer awareness about asthma, as well as improve patient communication with their GPs. |
| **What** |  |  |
| 3 | Materials: Describe any physical or informational materials used in the intervention, including those provided to participants or used in intervention delivery or in training of intervention providers. Provide information on where the materials can be  accessed (such as online appendix, URL) | Interactive Theatre:  The interactive theatre is delivered by an external partner, GLYPT (Greenwich and Lewisham Young People’s Theatre). GLYPT provides the actors who deliver the play and workshop with a script and organise rehearsals to ensure all actors are proficient in the content and objectives that are required of the workshop.  Asthma Workshops:  A script has been written for each workshop, which will be followed in all schools. Workshop providers will be part of the My Asthma in School team. All 4 workshops for young people with asthma use a range of props and other materials needed for the different workshop elements. A booklet is provided for each participant which includes information material about the topics addressed. The booklet is also used by the participants during the workshop to add information, e.g. tick the asthma triggers they are affected by. Each workshop uses a set of PowerPoint slides and general writing materials.  Further materials for workshop 1:   - 2 signs for each participant saying: Agree/disagree - Wall of fame: Envelope with laminated pictures of famous people who have asthma, Boards to sort these pictures. Stop watch to organise a race between groups sorting the pictures. - Asthma Balance Game (set up in advance): 2 balances with baby monsters that represent a young child, 2 x 4 bags with different toys representing things that can make developing asthma more likely, e.g. genes. There is always a light and a heavy version of each item. A heavy monster toy represents ‘chance’ and the fact that it is not always known why someone develops asthma. - Giant airway: giant prop of an airway, inflatable inner lining, and belts that can tighten on the outside, 2 barrels with putty representing additional mucus. Balls to send through the airways and bags to hold them in, an air pump to inflate and deflate the inner lining of the airways. Blue inhaler and brown inhaler props to demonstrate how the medication acts on the airways   Further materials for workshop 2:   - Interactive GP video:   - Film clips: ‘The Doctor will see you now’   - Doctors note sheets for every participant   - Asthma plan   - Peak flow meter and tubes for demonstration   - peak flow chart   - Visiting card to prepare notes for a health care visit - 10 trigger props to represent: pet, pollen, moulds (toxic mould spore), house dust mite, pollution (soot), colds and flu (common cold virus), cold air (snow globe), exercise (running figurine), smoking/second hand smoke (fake cigarette and vape pen), stress and emotions (stress ball), drugs (cannabis badge) - A2 trigger map to show where you usually encounter these triggers - 3 Asthma Dash board games   Further materials for workshop 3:   - Signs saying agree/disagree for everyone - Inhaler placebos in different shapes - 2 big inhaler props (blue and brown) that open to put in cards with statements about these inhalers - Mouth prop with   - Mouth to hang up   - Tube for trachea   - bag of balls,   - ball bin,   - big spacer prop to attach to mouth - Placebo inhalers and spacers for everyone - Apron with detachable symptom cards - Surgical mask to represent ‘can’t speak’ - Scarf to tie legs to represent ‘can’t walk’ - Role playing cards - Role play props: backpack with placebo and spacer, phone prop, headset   Further materials for workshop 4:   - Movie: Breaking the Jump (https://myhealthinschool.org/breaking-the-jump/) - Scenario cards - Problem solving video - Goal and steps postcards for everyone - Post bag - Certificates   Toolbox materials:   - Asthma dodge mobile app game - Asthma plan - visiting card for health appointment - Booster, quiz app |
| 4 | Procedures: Describe each of the procedures, activities, and/or processes used in the intervention, including any enabling  or support activities | As a first step schools are contacted and recruited to the project. Children with doctor diagnosed asthma in years 7 and 8 are identified in each school. In schools who receive the full intervention, all children of year 7 and 8 will take part in the ‘In Control’ theatre performance and theatre workshop. Children of years 7 and 8 with asthma will then take part in 4 one hour long workshops about asthma. They will receive a toolbox at the end of the workshops. The theatre only arm of the trial would initially only receive the interactive theatre as intervention.  During the trial, participating young people of all schools will fill out questionnaires to evaluate the effects of the intervention at several points in time: baseline questionnaire, repeat questionnaire directly after the intervention is delivered, as well as 3, 6, and 12 month follow-ups.  Schools who did not initially receive the full intervention are offered remaining intervention elements after the trial has finished. |
| **Who provided** |  |  |
| 5 | For each category of intervention provider (such as psychologist, nursing assistant), describe their expertise, background,  and any specific training given | The theatre and following interactive theatre workshop are run by the three professional actors who perform in the play. The actors rehearse the play, as well as the following workshop discussions with the theatre director at GLYPT. They are furthermore trained about asthma by members of the My Asthma in School Team, as well as by a clinician specialised in asthma, as part of their rehearsals. The theatre workshop discussions focus on relationships during the play, stigmatisation and peer awareness. The actors are advised to steer discussions away from detailed medical questions about asthma.  The asthma workshops are run by members of the My Asthma in School Project’s Learning and Outreach team. Team members are selected professionals with a university degree who have experience of working both in science, as well as in the communication of scientific topics. All providers use a detailed script to deliver the workshops. They are trained by more experienced members of the My Asthma in School team. All providers are educated about asthma and asthma management as part of their work within the My Asthma in School Project. |
| **How** |  |  |
| 6 | Describe the modes of delivery (such as face to face or by some other mechanism, such as internet or telephone) of the  intervention and whether it was provided individually or in a group | The intervention will be delivered face-to face. The theatre and theatre workshops are performed in front of the whole year group (years 7 and 8) with a maximum number of about 100 students in the audience. The workshops are delivered to young people with asthma from year7 and 8, with a maximum group size of 15. |
| **Where** |  |  |
| 7 | Describe the type(s) of location(s) where the intervention occurred, including any necessary infrastructure or relevant features | The intervention will be delivered in schools. The theatre team will perform usually in the assembly hall. The asthma workshops will be delivered in a class room. As the participants are under age, a member of staff needs to be attending at all times. |
| **When and How Much** |  |  |
| 8 | Describe the number of times the intervention was delivered and over what period of time including the number of sessions,  their schedule, and their duration, intensity, or dose | The full intervention was so far delivered once as part of a feasibility study. The asthma workshops in the feasibility study were delivered to 23 students in one group. The intervention will be delivered in 20 schools during the planned trial.  The theatre presentation lasts for approximately 40 Minutes followed by 30 minutes discussion.  The 4 asthma workshops are each one hour long and delivered within a week of the interactive theatre. |
| **Tailoring** |  |  |
| 9 | If the intervention was planned to be personalised, titrated or adapted, then describe what, why, when, and how | The participants of the asthma workshops fill out sections of the asthma booklet about their own asthma (e.g. which triggers and symptoms etc.). The workshops are interactive and therefore respond to individual concerns. Participants also set their own asthma goals in workshop 4. They write the goal down in their booklet and on a postcard. The research team is reminding them of the goal 1 month after the workshop by sending them their own postcard. |
| **Modifications** |  |  |
| 10 | If the intervention was modified during the course of the study, describe the changes (what, why, when, and how) | All intervention elements have been tested with young people. The intervention was furthermore reviewed both by learning, as well as clinical asthma experts, and a health psychologist. Changes were implemented accordingly. The feasibility study showed that some of the workshops were too long and that 23 participants for the workshops make on occasion discipline difficult. Some of the workshop elements were therefore shortened (e.g. less repeats). The maximum number of workshop participants was set at 15. |
| **How well** |  |  |
| 11 | Planned: If intervention adherence or fidelity was assessed, describe how and by whom, and if any strategies were used to  maintain or improve fidelity, describe them | The My Asthma in School team takes care that everyone involved in the intervention follows the study protocol and is appropriately trained. Centre of the Cell is providing basic training for the delivery of science workshops. The My Asthma in School workshops will be shown to new team members in run-throughs without participants. The new team members can then train to deliver these workshops in front of colleagues, before they take part in the workshop delivery in schools. There will always be at least one experienced member of the My Asthma in School project present at each school visits. Scripts, slides and props are in addition prepared to assure that the workshops are delivered in a consistent manor.  Fidelity will be assessed rating both competence and content of delivery. Records of adherence to procedures e.g. if postcards have been sent out in a timely manner will be examined and summarised as part of the pilot study by the My Asthma in School Project team. |
