# Supplementary figures and images for: Developing a theory-based multimedia intervention for schools to improve young people’s asthma: my asthma in school (MAIS)
Source: Pilot Feasibility Stud. 2020 Sep 2;6:122. doi: 10.1186/s40814-020-00670-6 (PMC7465390; doi:10.1186/s40814-020-00670-6)

## Supplement 2: Logic model

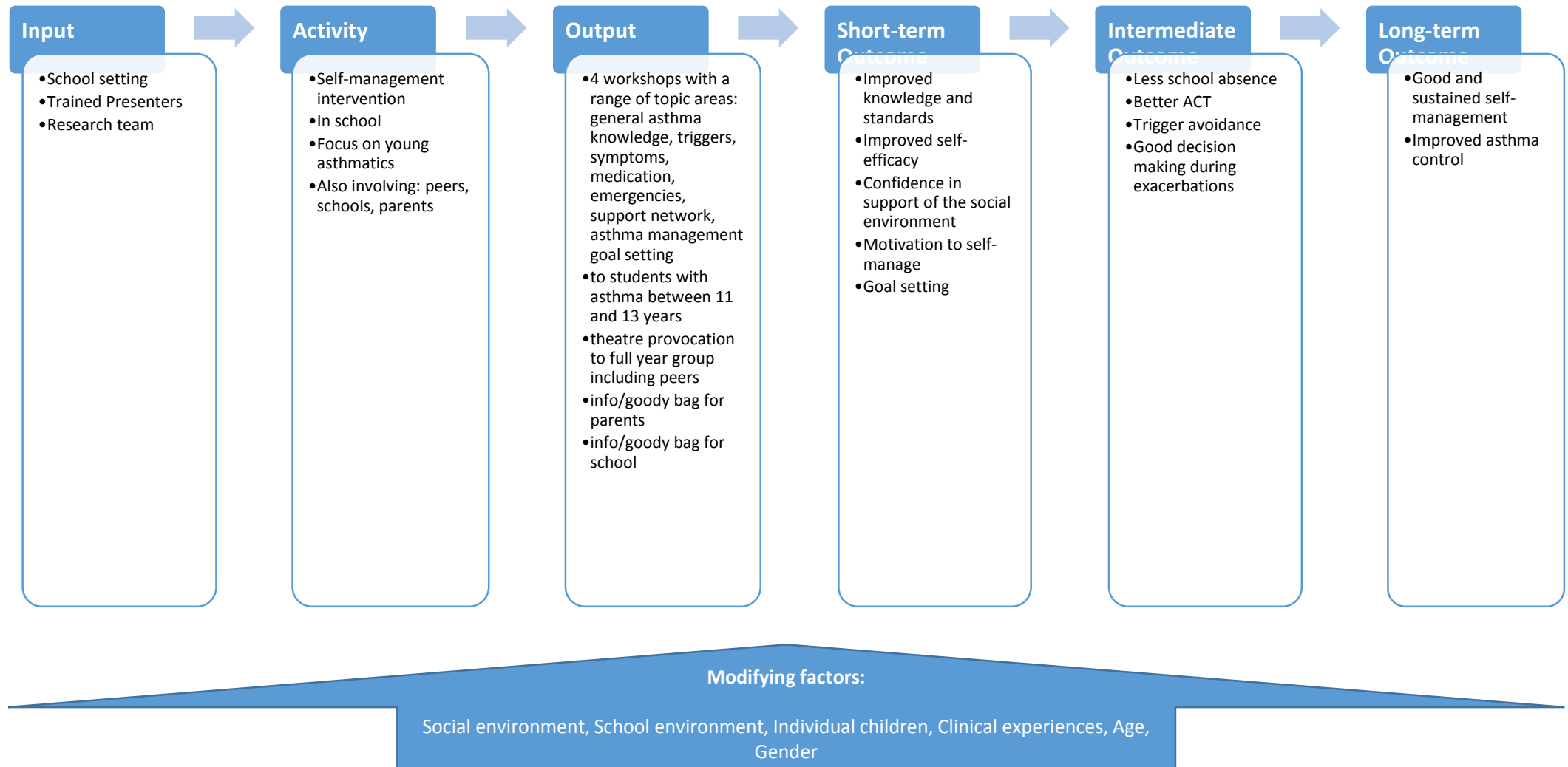

Supplement: Supplementary file 2 — Additional file 2:. Supplement 2: Logic model [file 40814_2020_670_MOESM2_ESM.pdf]
