# Supplementary material for: Developing a theory-based multimedia intervention for schools to improve young people’s asthma: my asthma in school (MAIS)
Source: Pilot Feasibility Stud. 2020 Sep 2;6:122. doi: 10.1186/s40814-020-00670-6 (PMC7465390; doi:10.1186/s40814-020-00670-6)
Supplement: Supplementary file 3 — Additional file 3:. Supplement 3: Elements of the intervention, behavioral targets related to them, as well as BCTs addressed by the element [file 40814_2020_670_MOESM3_ESM.docx]

## Supplement 3: Elements of the intervention, behavioral targets related to them, as well as BCTs addressed by the element

|  | ***Behavioral targets*** | ***Application to Intervention*** | ***BCT*** |
| --- | --- | --- | --- |
| ***In Control Theatre (full year group 7 or 8)*** | **MAIN TARGETED BEHAVIOR: A) Adherence to medication** |  |  |
|  | F3. Awareness and acceptance of asthma (including in non-asthma peers)  A12. Understanding what it means to live with asthma (including in non-asthma peers)  A11. Competent talking about asthma  F7. Appreciation of possibilities and limitations of a life with asthma  F5. Acceptance that asthma is part of them | **In Control Interactive Theatre**: play for the whole year group about the social impact of asthma followed by an interactive workshop during which the audience discusses solutions to the social situation with the actors. Topics include:   - the embarrassment of using a spacer in front of others, - how hard it can be to have asthma as a young person, - how difficult it might be to admit to asthma in social situations, - how dangerous it may be to deny having asthma. | 3.1 Social support  5.1 Information about health consequences  5.3 Information about social and environmental consequences  **5.2 Salience of consequences**  6.1 Demonstration of the behavior  6.3 Information about others approval  9.1 Credible source  **12.2 Restructuring the social environment**  13.2 Framing/reframing |
| ***Workshops - general*** |  |  | **9.1. credible source (trained team from university research trial)**  **X.1 Setting social context to change** |
| ***Workshop 1: What is Asthma? Who has asthma? (young people with asthma year 7 or 8)*** | **MAIN TARGETED BEHAVIORS: A) Adherence to medication & F) Empowerment to self-manage** |  |  |
|  | F5. Acceptance that asthma is part of them  F6. Appreciation that asthma is very common  A11. Competent talking about asthma | **Asthma is…:** quiz during which they compare their own beliefs about asthma with the statements from other young asthma sufferers | 8.1. Behavioral practice/rehearsal (A11: competent talking about asthma) |
|  | F5. Acceptance that asthma is part of them  F6. Appreciation that asthma is very common  F7. Appreciation of possibilities and limitations of a life with asthma | **Wall of fame**: Game around celebrities who have asthma. You can do anything in your life, providing examples of ‘cool’ and successful people who have asthma. Including statements by some celebrities about their asthma | 6.2. Social comparison  9.1. Credible source – asthma statements by some of the celebrities |
|  | F1. General understanding about asthma  A11. Competent talking about asthma  F2. Knowledge why someone develops asthma | **Asthma Balance Game**: why does someone develop asthma, interactive showcase | 4.3. Re-attribution (regarding causes of asthma) |
|  | A3. Understanding of how different inhalers function  A5. Awareness of the differences between long-acting corticosteroid inhalers and short-term acting SABA inhalers  A13. Awareness of the importance of medication adherence  F1. General understanding about asthma | **Giant Airways** (demonstration of healthy/asthmatic airways, as well as effects of medicine on the airways), interactive showcase. | 4.1. Instruction on how to perform the behavior – using inhalers  4.2. Information about antecedents – triggers  5.1. Information about health consequences – regarding triggers  **5.2 Salience of consequences** (regarding breathing with asthma and usage of medication with inflated size of asthmatic airways and inhalers)  11.1 Pharmacological support (encourage adherence to drugs) |
|  | A11. Competent talking about asthma | **Define Asthma**: Discuss with your neighbour how you would describe asthma to someone else. | 8.1. Behavioral practice/rehearsal – talking about asthma |
|  |  |  |  |
| ***Workshop 2: Symptoms and Triggers (only young people with asthma, year 7 or 8)*** | **MAIN TARGETED BEHAVIORS: A) Adherence to medication, C) Triggers & E) Communication with healthcare professionals** |  |  |
|  | A7. Appreciation of different asthma symptoms and symptom severity | **Symptoms Intro**: Discussion and demonstration about typical symptoms of asthma, interactive showcase | 5.1. Information about health consequences  4.1 Instruction on how to perform the behavior (recognizing/ identifying symptoms) |
|  | A3. Understanding how different inhalers work  A5. Awareness of the differences between long-acting corticosteroid inhalers and short-term acting SABA inhalers  A11. Competency talking about asthma  A13. Awareness of the importance of medical adherence  E1. Appreciation of good communication with healthcare professionals  E2. Understanding of the role of a healthcare professional  E3. Proficiency in methods of preparation for a visit with a healthcare professional | **GP consultation**: Film clips ‘The doctor will see you now!’ of 2 GP consultations during which participants take the role of the GP and take notes in her stead. This is followed by a discussion about effective communication with health care professionals. Furthermore, usage of an asthma plan and preparation of a healthcare visit is discussed in detail (an asthma plan and a GP visiting card is provided in their resource pack for them to take to their healthcare professional) | **1.2. Problem solving** (how to best prepare and behave during healthcare visits)  1.4. Action planning (visiting card, regular check-ups)  4.1. Instructions on how to perform the behavior (advise how to prepare for a healthcare visit and how to behave during visit)  *4.2. Information about antecedents (e.g. triggers)*  5.1. Information about health consequences (discussion about how the behavior of the two patients translates into differences in medical help)  5.2 Salience of consequences (film)  5.3 Information about social and environmental consequences  6.1. Demonstration of the behavior (communication)  7.1. Prompts/cues  *9.1. Credible source*  *11.1 Pharmacological support (encourage adherence to drugs)*  11.3 Conserving mental resources (using a visiting card instead of memorising)  12.5 Adding objects to the environment (handing out calling cards and asthma plan)  13.2. Framing/reframing (behavior from viewpoint of GP)  16.3. Vicarious consequences (discussion how much more patient B gets out of consultation compared to patient A) |
|  | A6. Comprehension of what is meant by well controlled asthma  A8. Knowledge how a peak flow meter can be used to monitor symptom severity | **Peak Flow Interactive**: Using the peak flow meter is demonstrated a volunteer and the concept of symptom monitoring through peak flow is introduced. Participants are encouraged to measure peak flow at home to monitor symptoms. (They will get a peak flow chart in their resource pack, as well as information about where to get a peak flow meter) | 2.6 Biofeedback  **4.1 Instruction on how to perform the behavior** (using a peak flow meter to monitor asthma severity)  6.1 Demonstration of the behavior  12.5 Adding objects to the environment (peak flow chart) |
|  | A6. Comprehension of what is meant by well controlled asthma | **Good Control**: Symptoms of several patients are discussed and participants are prompted to rate their asthma control. Their answers are subsequently discussed. | **4.1 Instruction how to perform a behavior** (assessing levels of asthma control)  6.1 Demonstration of the behavior (regarding assessment of asthma control)  *8.1 Behavioral practice/rehearsal (regarding assessment of asthma control)* |
|  | C1. Understanding about different triggers and how to mitigate their effect  C2. Proficiency in problem solving skills for difficult social situations related to triggers | **Trigger Map** (introduce and place location of triggers on local map, discuss how can they be mitigated), work with partner followed by discussion about each trigger | 3.2 Social support (practical) – parents can help with cleaning bedding etc.  **4.1 Instructions on how to perform the behavior** (Mitigating the effect of triggers on their asthma)  4.2 Information about antecedents  5.2 Salience of consequences (e.g. through big props)  5.3 Information about health consequences  7.5 Remove aversive stimulus  11.2 Reduce negative emotions (advise on stress management)  12.1 Restructuring the physical environment  12.2 Restructuring the social environment  12.3 Avoidance/reducing exposure to cues for the behavior  7.1 Prompts/ cues (set pollution alarm)  12.5 Adding objects to the environment |
|  | F1. General understanding about asthma  A11. Competent talking about asthma  A2. Awareness of situations in which they could forget their medication  A3. Understanding of how different inhalers work  A5. Awareness of the differences between long-acting corticosteroid inhalers and short-term acting SABA inhalers  C1. Understanding about different triggers and how to mitigate their effect  C2. Proficiency in problem solving skills for difficult social situations related to triggers  A13. Awareness of the importance of medication adherence  F5. Proficiency in problem solving skills for difficult social situations related to asthma  F8. Appreciation of possibilities and limitations of a life with asthma | **Asthma dash board game**: the game introduces a range of common triggers for asthma symptoms. You are more likely to win the game if you avoid the triggers or if you prevent symptoms by taking your corticosteroid inhaler. The game furthermore quizzes you about general asthma understanding. You get points for the right answer and behavior related to regular usage of corticosteroid inhalers. Several other concepts are introduced, e.g. that asthma changes over time. The person with the highest number of points wins. | DURING THE GAME:  *2.2 Feedback on behavior (how well player’s aviator adheres to brown inhaler…)*  *3.1 Social support (unspecified) – helping someone with asthma attack, help other players to get their inhaler*  *4.1 Instruction on how to perform the behavior (e.g. adherence)*  **5.2 Salience of consequences**  5.3 Information about social and environmental consequences  *6.2 Social comparison*  *7.1 Prompts/cues*  *7.5 Remove aversive stimulus (give away pet)*  *8.1 Behavioral practice/ rehearsal*  *9.3 Comparative imagining of future outcomes*  *10.3 Non-specific reward*  *10.11 Future punishment (you will sit out if you did not take your inhaler)*  *14.2 Punishment (losing brown inhaler or points when not managing asthma well)*  DESCRIPTION OF GAME RULES:  *4.2 Information about antecedents*  5.1 Information about health consequences  *11.1 pharmacological support (there are some medicines that help with pollen allergy)*  *11.2 Reduce negative emotions (advise on stress management)*  *12.1 Restructuring the physical environment (treat mold in home, wash bedding)* |
| ***Workshop 3: Medicines and Emergencies (only young people with asthma, year 7 or 8)*** | **MAIN TARGETED BEHAVIORS: A) Adherence to medication, B) Inhaler technique & D) Emergency response** |  |  |
|  | A3. Understanding of how different inhalers work  A4. Knowledge what to do in case of side effects | **Medication Myth Buster**: participants judge existing myths about medication, interactive presentation and discussion. Their answers are discussed. Participants are advised to inform their GP/clinician if they experience unpleasant side-effects with their medication | 5.1 Information about health consequences  **9.2 Pros and cons**  11.1 Pharmacological support (encourage adherence to drugs) |
|  | A5. Awareness of the differences between long-acting corticosteroid inhalers and short-term acting SABA inhalers | **Inhaler Shuffle**: participants sort statements about corticosteroid and SABA inhalers, group work and discussion | 5.3 Information about health consequences  11.1 Pharmacological support (encourage adherence to drugs) |
|  | B1. Proficiency in the correct inhaler technique  B2. Understanding how a spacer acts and what the benefits are of using a spacer  B3. Knowledge about how to acquire a spacer | **The big mouth:** Several participants demonstrate with a ball game how inhaler medication can end up at the back of the throat with incorrect inhaler technique. The information is reinforced by showing X-rays where the medication ends up. The presenter then educates the participants about the correct ‘puffer technique’ (without spacer). Several participants then demonstrate with a ball game how medication intake can be improved by using a spacer. The information is reinforced by showing X-rays where the medication ends up, showcase and interactive game. Participants are encouraged to ask their GP/clinician for a spacer if they don’t have one.  showcase and interactive game | 4.1 Instruction on how to perform the behavior  4.3 Re-attribution (reasons why medicine might not be effective)  5.1 Information about health consequences  **5.2 Salience of consequences** (through gaming)  6.1 Demonstration of behavior  12.5 Adding objects to the environment (information how to get a spacer)  11.1 Pharmacological support (encourage adherence to drugs) |
|  | B1. Proficiency in the correct inhaler technique  B2. Understanding how a spacer acts and what the benefits are of using a spacer | **Puffer Partners**: the presenter goes through the steps how to use the inhaler with spacer. The participants practice the technique. | 2.2 Feedback on behavior – by other participant and facilitator  4.1 Instruction on how to perform the behavior  6.1 Demonstration of the behavior  **8.1 Behavioral practice/ rehearsal (inhaler technique)** |
|  | D1. Skilled in asthma emergency response | **Emergency role play:** Participants are first taught which steps to take in an asthma emergency. Participants then role play emergency response with some of them playing and some directing their actions. | 1.2 Problem solving  3.2 Social support (practical) – advise that they should involve several others in case of emergencies  4.1 Instruction on how to perform the behavior  5.1 Information about health consequences  8.1 Behavioral practice/rehearsal |
| ***Workshop 4: Support network and taking control*** | **MAIN TARGETED BEHAVIORS: A) Adherence to medication, F) Empowerment to self-manage** |  |  |
|  | F5. Acceptance that asthma is part of them  F7. Appreciation of possibilities and limitations of a life with asthma  F8. Appreciation that asthma is very common  A11. Competent talking about asthma | **Breaking the jump short movie**: watch the short movie about a girl with asthma who learned how to control and live with her asthma, followed by a discussion. | 4.1 Instruction on how to perform the behavior (taking control of their asthma)  *4.2 Information about antecedents*  5.1 Information about health consequences  *5.2 Salience of consequences (film)*  5.3 Information about social and environmental consequences  6.1 Demonstration of the behavior (telling her friends about it; doing what she wants to do)  9.1 Credible source  12.1 Restructuring the physical environment  **13.2 Framing/reframing** (from not being in control to being in control) |
|  | F8. Proficient in methods aimed to change their asthma themselves, including: Where to get support | **Support target**: Different healthcare professionals and their role in helping with asthma are discussed. The idea of a support network is introduced, including friends, family, or teachers. The participants then note down who the important people are who can help with their asthma. | 3.1 Social support (unspecified) |
|  | F8. Proficient in methods aimed to change their asthma themselves, including how to break down a problem in order to find the most effective solution | **The third option:** Short film about a boy with asthma at sport practice, he forgot his inhaler and the film opens the discussion about possible underlying reasons. The film is followed by a group discussion about problem solving. | ***1.2 Problem solving***  *4.1 Instruction on how to perform the behavior*  *5.1 Information about health consequences*  5.2 Salience of consequences (film)  *5.3 Information about social and environmental consequences*  6.1 Demonstration of the behavior  *7.1 Prompts/cues*  9.1 Credible source  *9.2 Pros and cons*  *9.3 Comparative imagining of future outcomes* |
|  | A10. Awareness of the importance of reminders, prompts and cues, as well as the ability to set them up | **What can I do**? Participants are prompted to talk to other about their asthma, for example to a health professional, a parent, or a friend. Physical prompts and phone reminders for medication are introduced, participants are encouraged to set these up. | 3.1 Social support (unspecified)  7.1 Prompts/ cues   - 1. Restructuring the physical environment |
|  | A11. Competent talking about asthma  B4. Proficiency in problem solving skills for difficult social situations related to spacers  F4. Proficiency in problem solving skills for difficult social situations related to   - - Ridicule,   - Communicating symptoms,   - Responsibility to self-manage | **Asthma solution**: discussion in pairs training to give advice for difficult social situations related to asthma, including ridicule about asthma. This is followed by a group discussion | 1.2 Problem solving  3.1 Social support (unspecified)  5.3 Information about social and environmental consequences  **9.2 Pros and cons** |
|  | F8. Proficient in methods aimed to change their asthma themselves, including:  How to break down a problem in order to find the most effective solution  A10. Awareness of the importance of reminders, prompts and cues, as well as the ability to set them up | **Setting Goals**: the participants use the problem solving method WOP (wish obstacle plan) and set their own goal for asthma management. They write down their ‘wish, obstacle, and plan’ for their asthma management in their workbook. Then they are asked to write a postcard to themselves with a goal for their asthma, as well as steps to reach it, which will be sent to them in about 1 month time to remind them of their goal. | 1.2 Problem solving  1.3 Goal setting (outcome)  1.4 Action planning  1.9 Commitment  7.1 Prompts/cues (sending a reminder) |
| ***Workshop Booklet*** |  |  |  |
|  | B3. Knowledge about how to acquire a spacer  Other areas from above are repeated | A booklet is used during 4 workshops in which participants are prompted to write down several notes, for example their own symptoms, triggers, support network and goal. The booklet will be given to the participants to take home and includes information on:   - All areas taught in the workshops - Information how to get a spacer - Information how to get a peak flow meter - General information with links to websites about asthma | 4.1. Instruction on how to perform a behavior  4.2 Information about antecedents  5.1. Information about health consequences  9.2 Pros and cons (e.g. inhaler statements)  11.1 Pharmacological support (e.g. there are some medicines that help with pollen allergy)  11.2 Reduce negative emotions (advise on stress management)  12.1 Restructuring the physical environment (treat mold in home, wash bedding) |
| ***Toolbox*** |  |  |  |
|  | Sustained learning | **Boost mobile game app**: Boost is an app for mobile devices quizzing about improved understanding and skills conveyed as part of the workshops. The quiz is introduced in the workshop and the link to the app is given to them to take home for sustained learning. | 4.1 Instruction on how to perform a behavior  4.2 Information about antecedents  5.1 Information about health consequences  5.3 Information about health consequences  7.5 Remove aversive stimulus  11.1 Pharmacological support  11.2 Reduce negative emotions (advise on stress management)  12.1 Restructuring the physical environment  12.3 Avoidance/reducing exposure to cues for the behavior  7.1 Prompts/ cues |
|  |  | **Asthma action plan** | 4.1. Instruction on how to perform a behavior |
|  | A3. Understanding of how different inhalers work  A5. Awareness of the differences between long-acting corticosteroid inhalers and short-term acting SABA inhalers  A7. Appreciation of different asthma symptoms and symptom severity  A13. Awareness of the importance of medication adherence | **Asthma dodge mobile app game**: Asthma dodge is an infinite runner computer game, which can be used as an app on smartphones and tablets.  During the game intro the effect of asthma on the airways is explained. The game introduces a range of triggers that need to be dodged in order to be successful in the game. If you meet a trigger your character’s asthma symptoms get worse (coughing, wheezing). The game introduces corticosteroid and SABA inhalers, and prompts you to take them at appropriate moments in the game.  The game app is introduced in the workshop and the link to the app is given to them to take home for sustained learning. | 4.1 Instruction on how to perform the behavior  4.2 Information about antecedents  **5.2 Salience of consequences (gaming)**  5.3 Information about social and environmental consequences  6.1 Demonstration of the behavior  *7.1 Prompts/cues*  7.7 Exposure  *8.1 Behavioral practice/rehearsal*  *8.3 Habit formation (repeatedly using the correct inhaler at an appropriate time within the game)*  *8.7 Graded task (first avoid a few triggers in level 1, then more in level 2 and 3)*  *10.3 Non-specific reward*  11.2 Reduce negative emotions (advise on stress management)  12.2 Restructure the social environment  12.3 Avoidance/reducing exposure to cues for the behavior  *13.2 Framing/reframing (from the perspective of the aviator)*  *14.4 Rewarding approximation*  *14.5 Rewarding completion* |
|  | B3. Proficiency in methods of preparation for a visit with a healthcare professional | **GP calling card** | 7.1. Prompts and cues |
|  |  | **Peak flow chart** | 12.5 Adding objects to the environment |

In total 163 Behavioural Change Techniques (BCTs) were identified for the intervention elements, including 40 different BCTs. 29 intervention elements were coded separately, including single elements from the workshops and the toolbox. In line with our objective to improve asthma self-management skills, the BCT applied most frequently within the intervention was 4.1, ‘Instruction on how to perform the behaviour’ with sixteen applications and nine applications of 6.1, ‘Demonstration of the behaviour’ and 8.1, ‘Behavioural practice/ Rehearsal’ with seven applications. To address participants understanding of the causes and consequences of optimal self-management there were eight applications within the intervention of 4.2, ‘Information about antecedents’, 5.1, ‘Information about health consequences’ (thirteen applications), 5.3, ‘Information about social and environmental consequences’ (nine applications). These were further reinforced with nine applications of 5.2, ‘Salience of consequences’ to reinforce the relevance of these consequences for the individual.

The number of BCTs applied within an element, between 1 and 18, reflects how many different approaches to behavioural change are included within one element. A larger number of BCTs may however not be a direct reflection of how effective an element might be in improving self-management behaviour. During coding it became evident that elements with several BCTs often have one or two overarching techniques (indicated in bold in the table above).

- The highest number of different BCTs have been coded for two games: **‘Asthma Dash’** board game (Workshop 2) with 18 BCTs, and **‘Asthma Dodge’,** mobile app game (Toolbox) with 17 BCTs. Both games encompass several sub-behaviours including avoiding triggers and adherence to medication. They both put a focus on the reinforcement of behavioural change through 5.2, ‘salience of consequences’. Other BCTs that were coded for both elements include for example 7.1. ‘prompts/ cues’, 8.1. ‘behavioural practice/ rehearsal’, and 10.3. ‘non-specific reward’.
- The **interactive ‘GP consultation’** video clips (Workshop 2), aimed to improve communication during healthcare visits, have been coded with 15 BCTs, with 1.2. ‘problem solving’ as the overarching technique.
- The main focus of the **‘Trigger map’** activity (Workshop 2) is to instruct on how to mitigate and avoid the negative effect of asthma trigger using oversized trigger objects and a giant map of locations where these triggers could occur. The accompanying discussion introduces a wide variety of coping strategies for different triggers. 12 different BCTs were coded to the ‘Trigger map’ activity.
- A short video called **‘The third option’** (Workshop 4) aims to teach about 1.2 ‘problem solving’ techniques through the experience of a young person with asthma, who is developing strategies for a situation when he forgot to take his inhaler. This video was coded with 10 BCTs (link to video: <https://vimeo.com/301864867>).
- **‘Boost’**, a mobile app game (Toolbox), which repeats a range of knowledge gathered during the workshops has been coded with 10 BCTs.
- The **‘Breaking the Jump’** short video (Workshop 4) (link to video: https://myhealthinschool.org/breaking-the-jump/) and **‘In Control’** theatre performance (link to theatre documentary video: https://myhealthinschool.org/in-control-documentary/) are both coded with 9 BCTs. Both of these are aimed at asthma awareness. Breaking the Jump shows the transition of a young person from not being well controlled to being well controlled and its main aim was therefore coded with 13.2. ‘framing/reframing’. The interactive ‘In Control’ theatre is performed for the whole year group and as such aims to ‘restructure the social environment’ (12.2.) for young people with asthma by creating empathy and understanding in their peers. The theatre furthermore aims to reinforce the importance of supporting young people with asthma, ‘salience of consequences’ (5.2.) was therefore applied as another main BCT for this intervention element.
